# Effectiveness of high-intensity laser therapy in the treatment of musculoskeletal disorders: A systematic review and meta-analysis of randomized controlled trials: Erratum

**DOI:** 10.1097/MD.0000000000014274

**Published:** 2019-01-25

In the article, “Effectiveness of high-intensity laser therapy in the treatment of musculoskeletal disorders: A systematic review and meta-analysis of randomized controlled trials”,^[1]^ which appeared in Volume 97, Issue 51 of *Medicine*, the corresponding author email should be shj5th@korea.ac.kr.
